# Computed tomographic reference ranges of intrathoracic caudal vena cava ratios in sedated adult dogs without cardiac, pulmonary, or hypovolemic disease

**DOI:** 10.1002/vms3.1510

**Published:** 2024-06-18

**Authors:** Jacqueline N. Sankisov, Lauren Newsom

**Affiliations:** ^1^ Department of Clinical Sciences, Carlson College of Veterinary Medicine Oregon State University Corvallis Oregon USA

**Keywords:** canine, caudal vena cava, computed tomography, radiography, right‐sided cardiac disease

## Abstract

**Background:**

Radiographic assessment of the intrathoracic caudal vena cava (CVC) is commonly used to evaluate hemodynamic status in veterinary patients without and with pulmonary, pericardial, or right‐sided cardiac diseases. Many of these patients are now commonly evaluated with computed tomography (CT) in both emergency and referral settings. Traditional radiographic ratios in dogs, particularly the CVC height/aorta height (CVC/Ao) ratio, are often extrapolated to CT in order to determine if the CVC is normal in size.

**Objectives:**

The first goal of this retrospective study was to create an objective measurement method to evaluate the size of the CVC via CT. The second goal was to report normal CVC ratio values in both sagittal and transverse CT images.

**Methods:**

The traditional lateral radiographic CVC ratios were extrapolated to similar ratios obtained from sagittal CT images in stable, sedated adult canine patients without known cardiac or pulmonary disease. Additionally, new methods of canine CVC ratios using transverse CT images were defined using vessel height and area. Mean, standard deviation, and 95% confidence intervals (CIs) of the CVC ratios in transverse and sagittal CT images were calculated to determine normal reference ranges.

**Results:**

Ratio agreement from observers of different skill levels was moderate to excellent. Sagittal CT CVC/Ao mean was 1.07 ± 0.17 with a CI of 0.71.42. The mean and CI of transverse CT CVC/Ao height and area were 1.14 ± 0.27 and 0.781.44 and 1.36 ± 0.59 and 0.641.94, respectively.

**Conclusions:**

Application of normal sagittal and transverse CT ratio values in canine patients with and without hypotension, pulmonary, pericardial, or right‐sided cardiac diseases is necessary to determine the clinical usefulness of these ratios.

## Graphical abstract

An objective method to evaluate the size of the canine intrathoracic caudal vena cava in both sagittal and transverse computed tomography images is described in addition to reporting caudal vena cava ratio reference ranges for adult canines without known cardiac or pulmonary disease.

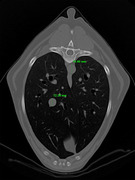

## 1 INTRODUCTION

The assessment of the intrathoracic caudal vena cava (CVC) via radiographs has long been used to evaluate hemodynamic status in canine and feline patients, in addition to evaluation of right‐sided heart failure in patients with primary pulmonary, pericardial, or right‐sided cardiac diseases (Lehmkuhl et al., 1997). The multitude of possible thoracic conformations and sizes among the canine population prohibits the use of an absolute CVC size measurement, and there can be wide variability in margin definition of the CVC which may create measurement error. Recent investigations have reported characteristics of anatomic CVC variations within the canine population (Bertolini et al., 2014; Choi et al., 2016; Ryu et al., 2019; Schwarz et al., 2009; Specchi et al., 2014). As a result, assessment of the intrathoracic CVC is often challenging, particularly for practitioners with less experience in evaluating thoracic radiographs and non‐imaging specialists. In a 1997 study published by Lehmkuhl et al., the CVC size was evaluated with ratios, including caudal vena cava height to aorta height (CVC/Ao), caudal vena cava height to length of the thoracic vertebrae above the tracheal bifurcation (CVC/VL), and caudal vena cava height to width of the fourth rib (CVC/R4) as measured in a left lateral thoracic radiograph. The CVC/Ao ratio has been widely used in the veterinary profession to evaluate the intrathoracic CVC in dogs radiographically. Lehmkuhl et al. established that a CVC/Ao <1 indicates a normal CVC size and a CVC/Ao >1.5 is highly suggestive of a right‐sided cardiac abnormality (Lehmkuhl et al., 1997). Increased CVC height in a lateral radiograph has been associated with right‐sided congestive heart failure from various congenital and acquired aetiologies, including pericardial diseases, pulmonic stenosis, tricuspid valve dysplasia or degeneration, dilated cardiomyopathy, and heartworm disease (Calvert & Thrall, 1982; Eyster et al., 1977; Losonsky et al., 2005; Suter & Lord, 1971). Small CVC and CVC/VL ratio have been associated with hypotension secondary to hypoadrenocorticism and hypovolemia (Melián et al., 1999). Importantly, a normal CVC/Ao ratio does not rule out the presence of right‐sided cardiac disease, right‐sided heart failure, or hypovolemia/hypotension (Lehmkuhl et al., 1997).

Patients with diseases that have been commonly assessed with radiographs are now often evaluated with computed tomography (CT) in many small animal clinical situations. Emergency centres and specialty referral centres use CT to evaluate patients with multiple diseases or traumatised body cavities, as a whole‐body staging tool, or when patients are large, fractious, and/or painful. In many cases, CT can be a timely way to obtain a large amount of patient information at one time, commonly under injectable and reversible sedatives. Currently, an objective method for evaluating the size of the intrathoracic CVC using CT in dogs is not available. The traditional radiographic CVC/Ao ratio is often extrapolated to CT as a means of evaluation. Defining a method to evaluate the size of the intrathoracic CVC on CT may improve real‐time evaluation and clinical decision making for many canine patients.

The first objective of this study was to perform the traditional CVC, Ao, rib, and vertebral measurements in sagittal CT images in sedated adult canine patients without cardiac or respiratory disease followed by calculation of the traditional ratios (CVC/Ao, CVC/VL, CVC/R4). The second objective of this study was to define new CVC measurements and ratios using transverse CT images. The sagittal and transverse CT ratios were used to define normal mean, standard deviation, 95% confidence intervals (CIs), and range. Lastly, interobserver agreement was calculated to assess whether skill level affected observer ability to perform the CT measurements.

## 2 MATERIALS AND METHODS

### 2.1 Selection and description of subjects

This retrospective study was conducted with medical records and picture archiving and communications system (PACS) from Oregon State University Carlson College of Veterinary Medicine, Veterinary Teaching Hospital, between December 2016 and December 2019. Use of patient data was approved by the Hospital Director. Initial inclusion criteria required DICOM thoracic CT imaging studies of dogs to be available in the PACS system. All studies were required to have thoracic sagittal and transverse CT planes in a high‐frequency algorithm available for review with a goal of 80 total patients. Once patients were confirmed to have the necessary imaging planes available, all components of the medical records were reviewed by a fourth year veterinary student and confirmed or rejected upon secondary evaluation by a board‐certified veterinary radiologist. Patients were excluded if there was a clinical concern for respiratory or cardiac disease documented in the medical record, including physical exam findings, documented diagnoses, and discharge instructions. Patients with a heart murmur, poor femoral pulse quality, inability to stand/evidence of collapse on history or presenting examination, abnormal mucous membranes, or capillary refill time greater than 2 s were excluded. Additionally, a rectal temperature less than 100°F or greater than 102.5°F, heart rate greater than 160 beats per minute, and resting respiratory rate greater than 24 respirations per minute were excluded. Bright, alert, and responsive patients panting on initial presentation were not immediately excluded, unless one of the other aforementioned criteria was met. Patients with a previous or current diagnosis of pulmonary (e.g. chronic bronchitis, pulmonary fibrosis, chronic coughing, pneumonia, pulmonary hypertension) or cardiac disease (e.g. congenital or acquired cardiac diagnoses, historical or existing heartworm disease) or with evidence of acute haemorrhage, hypovolemia, peritoneal fluid, or hypotension (e.g. haemoabdomen) documented in the medical record (history and physical exam findings), whether discovered during imaging evaluation or written in the discharge, were excluded. The age at the time of imaging, sex, neuter status, physical exam findings, and clinical diagnoses were recorded. Patient positioning for CT acquisition was chosen based on the primary disease focus for investigation and included either dorsal or ventral recumbency. All patients were either sedated or anaesthetised for the CT scan, and the medication protocol was decided by the primary anaesthesiologist and clinician and was not standardised. These protocols included, but were not limited to, dexmedetomidine, butorphanol, propofol, alfaxalone, methadone, hydromorphone, ketamine, diazepam, or inhalant anaesthesia (isoflurane or sevoflurane), any combination of which may have been used to achieve appropriate, safe anaesthetic plane for sufficient imaging quality. A web‐based viewing software (NovaRad, version 8.7.11; NovaRad Corporation) was used for all measurements, which were performed by a fourth year veterinary student and a board‐certified veterinary radiologist. All studies were anonymised using the NovaRad anonymisation software tool prior to performing measurements, which created a mixed number and letter labelling system and removed all identifying patient information from the study.

### 2.2 Data recording and analysis

All measurements were completed independently by a fourth‐year veterinary student and a board‐certified veterinary radiologist who were aware of the study design, exclusion criteria, and objectives. CT images of the thorax were acquired with patients positioned in either dorsal or ventral recumbency, depending on the needs of the primary disease workup. Non‐contrast CT images were acquired using a 64‐slice helical scanner (Toshiba Aquillion; Toshiba America Medical Systems, Inc) with the following parameters: 120 kVp, 200–400 mA, and pitch of 0.5–0.6 depending on the size of the patient. Images were reconstructed into 3‐mm slices, and measurements were made using a high‐frequency algorithm in a bone window.

**FIGURE 1:**
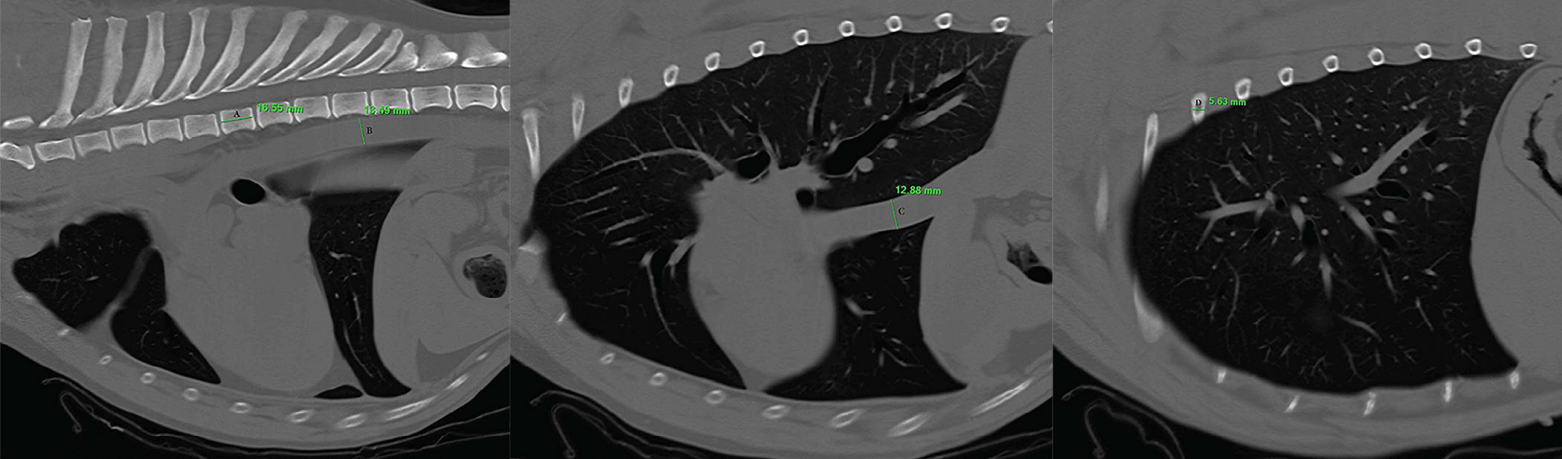
Sagittal thoracic computed tomography image demonstrating vertebral body length (A) at the level of the tracheal bifurcation, aortic height (B) measurement at the same rib space as the caudal vena cava height (C) measurement, and 4th rib width (D) measurements parasagittal to the spine. These measurements were performed in 80 sedated adult dogs without cardiac, pulmonary, or hypovolemic disease.

**FIGURE 2:**
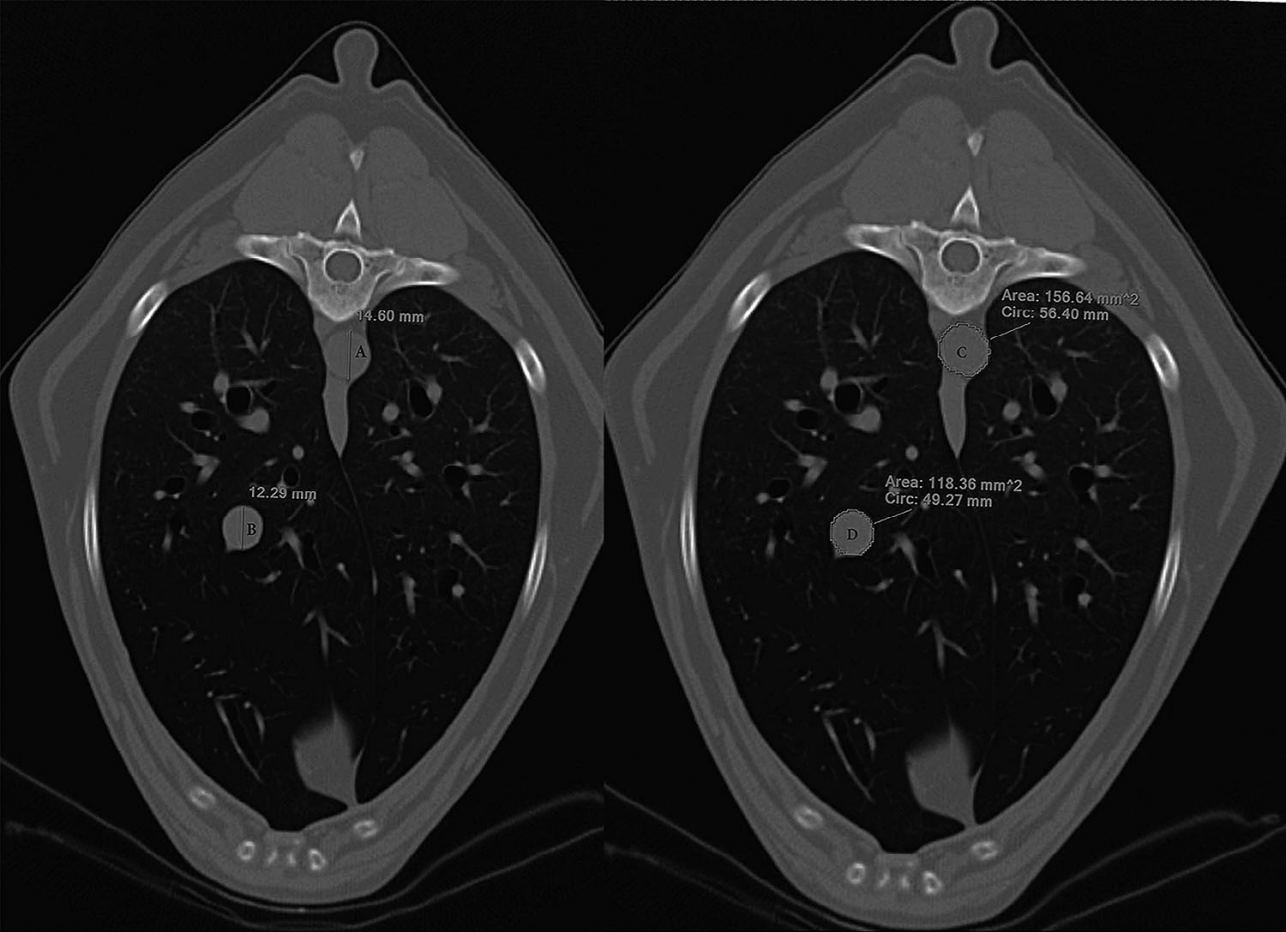
Transverse thoracic computed tomography images demonstrating aorta (A) and caudal vena cava height measurements (B) and circumferential area measurement of the aorta (C) and caudal vena cava (D). All transverse measurements were performed on the same slice, where the vessels were most round, well‐defined, and not in contact with either the heart or diaphragm. These measurements were performed in 80 sedated adult dogs without cardiac, pulmonary, or hypovolemic disease.

For each patient, the following measurements were performed on the sagittal CT images (Ao, CVC, VL, and R4; Figure 1) as previously described in a radiographic study by Lehmkuhl et al. The vessel measurements were taken at location with the least amount of motion artifact and with the vessel at maximal height, without overlapping the cardiac silhouette or diaphragm. The R4 measurements were performed in a parasagittal plane adjacent to the spine, measured cranial to caudal, when the vertebra was no longer present in the image. For each observer, the following sagittal CT ratios were calculated: CVC/Ao, CVC/R4, and CVC/VL. For the transverse measurements, a single transverse image was chosen where the CVC was maximal in height and most round in shape without overlapping the cardiac silhouette or diaphragm. This image was used to record the following height and area measurements (Figure 2):

Caudal vena cava height (CVCh): The height of the CVC was measured perpendicular to the long axis of the vessel.

Caudal vena cava area (CVCa): The area measuring tool was used to trace along the peripheral margin of the CVC.

Aorta area (Aoa): The area measuring tool was used to trace along the peripheral margin of the aorta.

**FIGURE 3:**
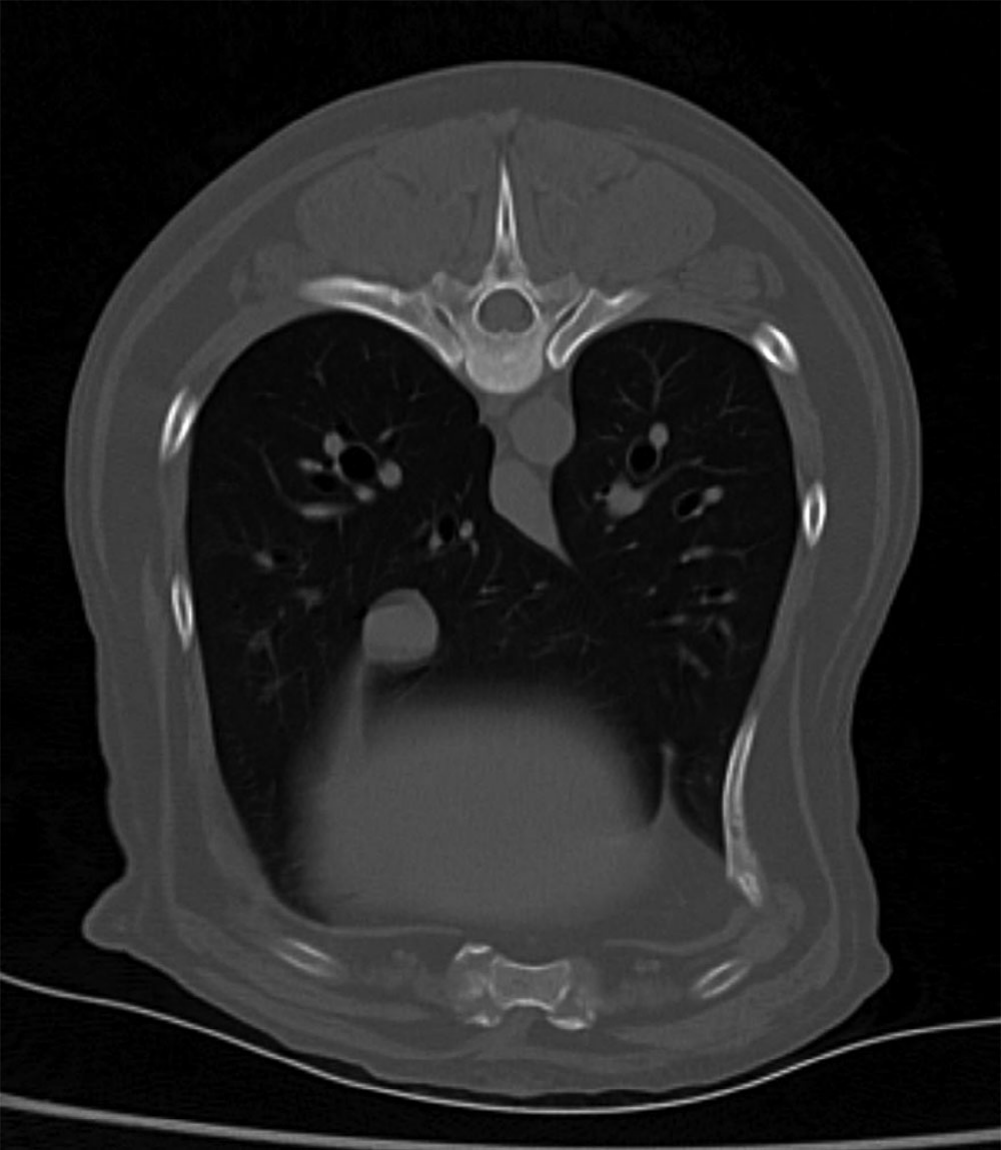
Transverse computed tomography image of the caudal thorax highlighting motion artifact that results in ill‐defined caudal vena cava margins.

Linear measurements were performed independently and recorded once by each observer for each patient. Area measurements were recorded three times and averaged for each observer before ratio calculation. For each observer, after the measurements were complete, the following transverse CT ratios were calculated: CVCh/Aoh and CVCa/Aoa. Attempts were made by both observers to use the part of the CVC with the least amount of motion artifact (Figure 3). Observers were allowed to use magnification, scroll, and window/level adjustments as necessary while obtaining measurements. Slice selection was not standardised between observers.

### 2.3 Statistics

Statistical tests were chosen and performed by an experienced professional statistician at our institution. Reference ranges, mean, standard deviation, and a 95% CI for the transverse CT and sagittal CT ratios were reported, and the measurements from both observers were averaged prior to statistical evaluation (Lin, 1989). Statistical analysis was performed with R‐4.1.2 and the reference intervals package. The normality of the data was tested using the Kolmogorov–Smirnov test, and screening for outliers was done using Horn's method and Tukey's interquartile fences on a Box–Cox transformation of the data when needed. For variables that were normal, 95% parametric intervals are reported. Additionally, mean and standard deviation were calculated from the averaged observer values for the CT ratios. Lin's (1989) concordance coefficient was used to calculate interobserver agreement for the sagittal and transverse CT ratios.

## 3 RESULTS

Eighty canine patients identified in a university hospital information system between 2016 and 2019 met inclusion criteria. Many dog breeds were represented, with Labrador Retriever (*n* = 15), Border Collie (*n* = 6), Australian Shepherd (*n* = 4), and Boxer (*n* = 4) being the most numerous. There were three or less of the following breeds: Golden Retriever, unspecified mixed breed, Labradoodle, Schnauzer, Great Dane, German Shepherd, Beagle, Alaskan Malamute, German Short‐Haired Pointer, Flat‐coated Retriever, Chow‐Chow, Norwegian Elkhound, Great Pyrenees, Catahoula Mix, Eskimo, French Bulldog, Jack Russel Terrier, Pembroke Welsh Corgi, Miniature Dachshund, Cockapoo, Siberian Husky, English Pointer, Staffordshire Terrier, German Shephard, Swiss Mountain Dog, Akita, Basset Hound, Shephard Mix, Blue Heeler, Belgian Malinois, Cardigan Corgi, Terrier Mix, Australian Cattle Dog, Irish Setter, Saint Bernard, and Puli. Median age at presentation was 8.4 years (range, 11 months to 14 years), and median weight was 30.5 kg (range, 6–61.5 kg). There were 43 castrated males, 30 spayed females, five intact males, and two intact females.

Initial presenting complaints were varied and typical for a tertiary referral institution, and the included patients were admitted for imaging via scheduled appointments through either the small animal surgery, oncology, or small animal internal medicine departments. No walk‐in or emergency and critical care department was available at our institution between 2016 and 2019. Presenting complaints included fever of unknown origin (*n* = 3), intervertebral disc disease (*n* = 2), liver mass (*n* = 2), lipoma (*n* = 2), fungal rhinitis (*n* = 2), and one of each the following: back pain localised to T7–T8, discospondylitis, coughing, brachycephalic obstructive airway syndrome, post‐operative adrenalectomy, laryngeal paralysis, acute nephritis, acute hepatitis, acute pancreatitis, acute gastroenteritis, primary hyperparathyroidism, epistaxis, polycythemia, ataxia, cutaneous thigh mass, insulinoma, testicular mass, jejunal mass, ear mass, nasal mass, cervical mass, reactive fibrovascular proliferation of the gingiva, axillary mass, vehicular trauma, and elongated soft palate. Many patients presented for staging, recheck, and/or metastasis check for diverse neoplasms, including osteosarcoma (*n* = 10), splenic hemangiosarcoma (*n* = 6), apocrine gland anal sac adenocarcinoma (*n* = 4), soft tissue sarcoma (*n* = 3), histiocytic sarcoma (*n* = 3), and one each of the following: cutaneous mast cell tumour, digital melanoma, cutaneous melanoma, oral fibrosarcoma, orbital myxosarcoma, undifferentiated sarcoma, nasal squamous cell carcinoma, digital squamous cell carcinoma, rectal carcinoma, oral melanoma, suspected T‐cell thymic lymphoma, urinary transitional cell carcinoma, chronic lymphoid leukaemia, prostatic carcinoma, retroperitoneal hemangiosarcoma, sebaceous gland adenocarcinoma, nasal adenocarcinoma, and oral malignant adenoma. All patients included in this study were clinical cases and were either sedated or anaesthetised for the CT scan with protocols determined by the primary clinician depending on the needs of each individual patient. No breath holds were performed during the imaging examination on either sedated or anaesthetised patients, as this is not standard protocol at our institution. Patients were positioned either is sternal or dorsal recumbency, depending on the needs of the imaging study for the primary disease evaluation.

After each observer independently performed the measurements in sagittal and transverse CT images and the ratios described above were calculated, Lin's concordance coefficient was used to calculate interobserver agreement for the sagittal and transverse CT ratios. The values are reported in Table 1. Values range from −1 to +1, and values near +1 indicate strong agreement between the observers, values near −1 indicate strong disagreement between the observers, and values near 0 indicate no agreement. These values were interpreted similar to Pearson's correlation coefficient, where values less than 0.20 indicate poor agreement, values between 0.20 and 0.80 indicate moderate agreement, and values greater than 0.80 indicate excellent agreement. All ratios had moderate to excellent agreement. Both of the transverse CT ratios (CVC/Ao ratios of height and area) had excellent agreement.

**TABLE 1:** Interobserver agreement (Lin's concordance coefficient) for the sagittal and transverse ratios calculated from computed tomography measurements performed in 80 sedated adult dogs without cardiac, pulmonary, or hypovolemic disease.

	Computed tomography agreement	
Caudal vena cava ratio	Sagittal	Transverse
CVC/Ao	+0.79	–
CVC/R4	+0.60	–
CVC/VL	+0.93	–
CVCh/Aoh	–	+0.84
CVCa/Aoa	–	+0.99

Abbreviations: CVC/Ao, sagittal caudal vena cava/aorta ratio; CVC/R4, sagittal caudal vena cava/4th rib ratio; CVC/VL, sagittal caudal vena cava/vertebral length ratio; CVCa/Aoa, transverse caudal vena cava area/aorta area ratio; CVCh/Aoh, transverse caudal vena cava height/aorta height ratio.

Lastly, the sagittal CT and transverse CT ratios were averaged across both observers, and the mean, standard deviation, 95% CI, and ranges were calculated and reported in Table 2. All variables passed the test for normality (*p*‐values for normal test >0.05). Outliers detected by the procedure were included in calculations of the CIs. The sagittal CVC/Ao mean was 1.07 ± 0.17 with a range of 0.71. The sagittal CVC/R4 mean was 2.62 ± 0.45 with a range of 1.74. The sagittal CVC/VL mean was 1.0 ± 0.17 with a range of 0.67. The mean of the transverse CT CVC/Ao height and area ratios were 1.14 ± 0.27 with a range of 1.37 and 1.36 ± 0.59 with a range of 2.71, respectively.

**TABLE 2:** Mean, standard deviation, 95% confidence interval, and range of the sagittal and transverse ratios calculated from computed tomography measurements performed in 80 sedated adult dogs without cardiac, pulmonary, or hypovolemic disease.

Caudal vena cava ratio	Computed tomography plane	Mean	Standard deviation	95% confidence interval	Range
CVC/Ao	Sagittal	1.07	±0.17	1.0–1.15	0.71
CVC/R4	Sagittal	2.62	±0.45	2.43–2.81	1.74
CVC/VL	Sagittal	1.0	±0.17	0.92–1.07	0.67
CVCh/Aoh	Transverse	1.14	±0.27	1.03–1.25	1.37
CVCa/Aoa	Transverse	1.36	±0.59	1.11–1.61	2.71

Abbreviations: CVC/Ao, sagittal caudal vena cava/aorta ratio; CVC/R4, sagittal caudal vena cava/4th rib ratio; CVC/VL, sagittal caudal vena cava/vertebral length ratio; CVCa/Aoa, transverse caudal vena cava area/aorta area ratio; CVCh/Aoh, transverse caudal vena cava height/aorta height ratio.

## 4 DISCUSSION

No objective method for clinical interpretation of the CVC size in dogs with CT is readily available. In this retrospective study, we extrapolate traditional radiographic measurements and report a new evaluation method and a range of ratio values for the CVC size in sagittal and transverse CT images for sedated, stable adult dogs without cardiac or pulmonary disease. Assessment of the intrathoracic CVC via CT is important in a variety of patients including trauma patients, patients with chronic illness or peritoneal fluid, and particularly patients with known cardiac or pulmonary diseases. With CT becoming more widely available, an objective method of evaluation of size will be useful to radiologists, general practitioners, and non‐imaging specialists alike.

Clinical extrapolation of the traditional radiographic ratio values to sagittal CT images is likely reasonable when comparing the CT ratio values to the previously reported radiographic ratios. As previously reported by Lehmkuhl et al., radiographic CVC/Ao ratio of <1.0 represents dogs without right‐sided cardiac disease, and radiographic CVC/Ao ratio >1.5 is suggestive of a right‐sided cardiac abnormality (Lehmkuhl et al., 1997). In our study, the sagittal CT CVC/Ao mean was 1.07 ± 0.17 with a range of 0.7, which fits within the previously published parameters for a normal CVC/Ao ratio and indicates that a right‐sided cardiac abnormality is unlikely. Patients with a radiographic CVC/R4 ratio <2.25 were reportedly highly unlikely to have right‐sided heart failure, and a radiographic CVC/R4 ratio >3.75 was indicative of right‐sided heart failure (Lehmkuhl et al., 1997). In our study, sagittal CVC/R4 mean was 2.62 ± 0.45 with a range of 1.74, which supports the clinical conclusion that our patients were not in right‐sided heart failure. Patients with a radiographic CVC/VL ratio <0.8 represents dogs that are unlikely to have right‐sided heart failure, and a radiographic CVC/VL ratio >1.3 may indicate right‐sided heart failure (Lehmkuhl et al., 1997). In our study, the sagittal CVC/VL mean was 1.0 ± 0.17 with a range of 0.67, which is also supportive of the conclusion that our patients were not in right‐sided heart failure. Comparison of the mean, standard deviation, and CI values reported in this study with the results reported by Lehmkuhl et al. is not possible as this information is not available. Similar to what was described with extrapolation of the radiographic vertebral heart scale to CT (Timperman et al., 2021), inherent differences between the two modalities may result in different measurements. Radiographs are a summation modality, which allows evaluation of an anatomical structure as a whole, while CT allows evaluation of individual slices with improved margin definition and greater accuracy for detail as compared to radiographs. Radiography also has existing magnification artifact. It is expected that CT measurements would be smaller than radiographic measurements due to these differences. Given the overall small absolute size of the measurements performed, statistical significance may not translate to clinical significance. Extrapolation of the traditional radiographic ratio values to sagittal CT images is likely reasonable; however, at the high end of the size range, a false‐negative diagnosis of normal CVC size on CT is possible.

Not surprisingly, the transverse CVCh/Aoh ratio value is larger than the sagittal CVC/Ao ratio value (Table 2). This likely reflects the observer's ability in the transverse plane to choose a single slice where the vessels were most round and tallest in height. The CVC and Ao have a variation in path from the diaphragm to the heart, often coursing mildly obliquely from caudodorsal to cranioventral. This may result in mildly oblique transverse height measurements of both vessels, which may be exaggerated or minimised depending upon patient body morphology differences. Additionally, neither the CVC nor Ao is parallel to the parasagittal imaging plane of the CT scan, which may have resulted in smaller height measurements in the sagittal plane images.

Images in a high‐frequency algorithm and bone window were chosen to create the best margin definition of the CVC and Ao during measurement. A soft tissue window used with a high‐frequency algorithm results in exacerbation of inherent image noise. A low‐frequency algorithm inherently smooths the edges of a structure, which can result in difficulty determining exact margination of the structure being measured. Additionally, due to partial volume averaging, gas attenuation adjacent to the CVC and Ao in a soft tissue window will cause the peripheral margins of the vessel to be averaged with gas, resulting in lower attenuation values. This could result in both ill‐defined vessel margin definition and overall artefactual reduction in vessel size.

Clinical utility of imaging measurements can hinge on whether different observers can perform measurements similarly. In this study, the interobserver agreement between a fourth year veterinary student and a board‐certified veterinary radiologist for all CT ratios performed was moderate to excellent. The ratio with the poorest interobserver agreement was the CVC/R4 ratio at 0.6. The R4 measurements were measured cranial to caudal in a parasagittal location where the vertebra was no longer present in the image. The specific image for the R4 measurement was not standardised between observers, and this portion of the rib is naturally curved in shape, which resulted in occasionally challenging linear measurements and only moderate agreement. The overall moderate to excellent agreement between observers with varying levels of experience potentially makes these methods more clinically applicable than measurements that need specific software or advanced skill to obtain.

Electrocardiogram (ECG) gating of the CT studies was not performed nor sought out during retrospective assessment of the studies, in an additional attempt to be widely clinically applicable. Many facilities with CT capabilities do not have the software or staff available to effectively execute an ECG‐gated study. As previously reported, there is little difference in the vertebral heart scale as measured in gated and non‐gated CT scans (Timperman et al., 2021). Additionally, both sagittal and transverse image sets were evaluated to create ratios that can be used by veterinarians with and without the ability to create sagittal CT reconstructions adequate for accurate measurements.

Limitations of our study include primarily the retrospective design, which resulted in non‐standardised sedation and anaesthetic protocols, potential for variable respiratory phase at imaging, use of intravenous treatments, non‐standard positioning between patients, clinical patients with various non‐cardiac and non‐pulmonary diagnoses, non‐ECG‐gated CT studies, and lack of a full echocardiographic workup in all patients. Evaluation of the included patient's entire medical record was retrospective; however, if any question of existing cardiac, respiratory, or hemodynamic abnormalities was documented in the medical record or imaging, the patient was excluded. Non‐standardised positioning included patients placed in either sternal or dorsal recumbency. Abdominal venous compression may affect venous return depending on patient size, further influencing CVC size. Given that sedation, anaesthesia, positioning, and monitoring protocols were determined by the primary disease workup, primary clinician, and the anaesthesiologist, phase of respiration and blood pressure measurements were also not standardised. This includes the possibility of variations in central venous pressure due to the use of dexmedetomidine sedation and the lack of inspiratory/breath‐hold imaging. The authors acknowledge the limitations in comparison of data, as sedation with dexmedetomidine has recently been associated with transient gallbladder wall thickening and peritoneal effusion in some dogs undergoing abdominal ultrasonography (Seitz et al., 2021), likely due to variations in Starling's forces. Non‐sedated CT imaging in stable canine patients is not performed regularly at our institution, as this is often not achievable, would increase motion artifact, and creates issues related to patient safety. Patients clinical for hypovolemia or hypotension (e.g. collapse, tachycardia, poor pulse quality, increased CRT, pale mucous membranes) may be imaged without sedation due to their disease status; however, this patient population was excluded in the initial phase of this study. The authors feel the information reported here remains clinically valuable despite these variables. A prospective study performed with healthy, medium‐sized dogs undergoing anaesthesia with a breath hold may create a seamless research study; however, it would not represent the real dogs seen clinically at our hospital and many others. The included cases are representative of the reality of stable patients seen in veterinary practice today. CT images had varying degrees of motion artifact due to respiratory motion, which resulted in difficulty measuring the CVC in some patients. Although slice selection for measurements was not standardised between observers, measurement agreement between the observers of different skill levels remained moderate to excellent.

An objective method to evaluate the size of the intrathoracic CVC in both sagittal and transverse CT images is described herein, in addition to reporting CVC ratio reference ranges for adult dogs without cardiac or pulmonary disease. Ultimately, in order to determine the clinical usefulness of these ratios, application of the ratios in dogs with various thoracic and abdominal diseases is necessary before widely recommending the use of these ratios in everyday patient management.

## AUTHOR CONTRIBUTIONS

**Lauren Newsom**: Conceptualisation; data curation; formal analysis; investigation; methodology; project administration; resources; software; supervision; validation; visualisation; writing—original draft; writing—review and editing. **Jacqueline Sankisov**: Data curation; Formal analysis; Investigation; Methodology; Project administration; Resources; Software; Supervision; Validation; Visualisation; Writing—original draft; Writing—review & editing.

## CONFLICT OF INTEREST STATEMENT

The authors declare no conflicts of interest.

## FUNDING INFORMATION

The authors received no specific funding for this work.

### ETHICS STATEMENT

The authors of this article confirm that the ethical policies of this journal, as noted in the author guidelines, in addition to ethical handling of patient data, have been adhered to.

### PEER REVIEW

The peer review history for this article is available at https://publons.com/publon/10.1002/vms3.1510.

## Data Availability

The data that support the findings of this study are available on request from the corresponding author. The data are not publicly available due to privacy or ethical restrictions.
